# The Influence of Parenting Style and Time Management Tendency on Internet Gaming Disorder among Adolescents

**DOI:** 10.3390/ijerph17239120

**Published:** 2020-12-06

**Authors:** I-Hua Chen, Zeng-Han Lee, Xiao-Yu Dong, Jeffrey Hugh Gamble, Hung-Wei Feng

**Affiliations:** 1 School of Education Science, Minnan Normal University, Zhangzhou 363000, China; aholechen@gmail.com; 2 College of Teacher Education, Wenzhou University, Wenzhou City 325035, China; zenghan88@gmail.com; 3 College of Education, Qufu Normal University, Qufu 273165, China; noodlelin0406@gmail.com; 4 Department of Foreign Languages, National Chiayi University, Chiayi 62103, Taiwan; 5 College of Education Science, Yulin Normal University, Yulin 537000, China

**Keywords:** parenting styles, time management tendency, online game addiction, internet gaming disorder, adolescence

## Abstract

The problem of adolescent online gaming addiction is related to individual-level characteristics and the influence of the family environment. The present study explores the potential role of adolescents’ time management tendency in mediating the relationship between parenting style and adolescent internet gaming disorder (IGD). Responses from a total of 357 Chinese high school students were collected for a Pathological Video-Game Use Questionnaire, Simplified Parenting Styles Scale, and Time Management Tendency Scale. Overall, participants reported moderate use of online games (Mean = 1.41; SD = 0.41), lower than the median value of 2 on a three-point scale. In terms of the mediating role of adolescents’ time management tendency, full meditation was observed for the relationship between the parenting style factor of “parents’ emotional warmth” for both mothers and fathers and internet gaming disorder. The results highlight the benefits of emotional warmth in supporting self-efficacy, self-control, and autonomy through the promotion of time management, which is an important protective factor for IGD and can serve as a mediating personality variable. Although non-significant in the complete model, over-protection and rejection by parents should also be cautiously considered as potential risk factors related to addiction.

## 1. Introduction

### 1.1. Effects and Prevalence of Internet Gaming Disorder (IGD)

Despite the conveniences of the Internet for studying, entertainment, or other daily life activities, the psychological risks associated with inappropriate internet use are receiving increasing attention. Clinical diagnosis of internet addiction includes users’ excessive use, feelings of withdrawal when access is unavailable, and other negative psychological effects, including poor academic achievement, exhaustion, and social isolation [1]. Other negative effects that serve as criteria for diagnosing “internet use disorder” as an impulse-related disorder include the development of tolerance (and the need for increased use to feel satisfied), and harmful physical, psychological, and social effects [2]. One element of internet addiction that is most frequently observed among adolescents and school-aged children is online game addiction [3,4]. Among the negative effects resulting from online game addiction are decreased performance on work and study tasks, interpersonal relationship difficulties, and lower psychological well-being [5]. In fact, the prevalence and severity of online game addiction internationally, in addition to increased attention to the assessment and prediction of pathological online gaming behaviors, has led to the inclusion of Internet Gaming Disorder (IGD) in the Diagnostic and Statistical Manual of Mental Disorders (DSM-5) [6]. The nine criteria for diagnosing IGD by the DMS-5 include: preoccupation, withdrawal, tolerance, difficulty reducing or stopping, continued game play despite problems, giving up other activities, using games to escape adverse moods, deceiving or covering up game use, and risk or loss of relationships or opportunities [7].

In the context of the present study, according to the latest survey by the China Internet Network Information Center (CINIC), in the first half of 2018, the number of online game users in China reached 486 million [8]. Among these users, 25.1% were students in high schools or vocational schools [8]. Compared to other regions, adolescents in China have higher reported rates of IGD, with recent studies finding a prevalence of 3.5 to 17% from a systematic review of Chinese research reports [9] and an average of 13% from a cross-sectional survey published in 2020 [10]. Reported rates of online game addiction for Chinese adolescents is higher than the international average, as reported by other recently published studies, ranging from 1.96 to 5.7% [11,12,13]. As adolescents are more actively involved in internet gaming activities as compared to other age groups [14], studies increasingly report cases of Chinese high school students dropping out of school due to IGD and suffering from a variety of negative social and psychological impacts as a result [15,16]. Outside of academia, media outlets have also responded to the prevalence of internet game addiction by criticizing the use of online games by adolescents and publishing reports to raise public awareness of the dangers of online games [17,18]. Increased scrutiny on the prevalence and negative effects of IGD has naturally led to an evaluation of the factors influencing online gaming behaviors.

### 1.2. Factors Influencing Adolescents’ Internet Gaming Disorder

A variety of factors have been investigated as predictors or risk factors for internet addiction, among which are social and family environmental factors and personality factors. In a recent and well-cited systematic review evaluating cross-sectional and longitudinal studies related to IGD, Mihara and Higuchi [19] highlighted the potential role of familial factors, stating “family and parenting factors” as the only socially related category of risk or protective factors. Mihara and Higuchi [19] also found that “personality” appeared as a significant factor in the literature on risk factors for addictive online gaming, stating that the personality factor of “perceived behavioral control was found to be the most important factor in predicting problematic video-game behavior” and that “a client’s perceived lack of control over gaming may be a simple but useful measure to evaluate risk of future problem play” (p. 439). The important role of self-efficacy, an indicator of an individual’s belief in their ability to execute specific tasks and a behavioral determinant has been shown by recent research to be clearly related to excessive or addictive internet use and IGD [20,21]. The following sections highlight the potential role of parenting style (as a family and parenting factor) and time management tendency (as a personality factor) as risk or protective factors in terms of Internet Gaming Disorder.

#### 1.2.1. Family and Parenting Factors: Parenting Style

The relationship between parenting and adolescents’ IGD has been investigated by prior studies, including factors such as parental attachment and attitude (acceptance vs. rejection) [3], parenting styles (including permissive, authoritarian, and authoritative) [22], parental monitoring [23], online security measures [24], parental rearing [25], and parental bonding [24,26]. Based on the considerable amount of research related to the role of parents in adolescent online gaming behaviors, it is reasonable to assume that parenting can serve as a significant predictive variable in terms of adolescents’ potential IGD. In fact, a meta-analysis of the relationship between parenting style and Chinese adolescent internet addiction, based on 232 published studies, found a moderate negative correlation between positive indicators (such as emotional warmth and understanding) and internet addiction, suggesting that positive parenting can reduce the severity of internet addiction [27]. In fact, the direct effect of mothers’ emotional warmth was found to decrease the risk of pathological internet use, while the direct effect of fathers’ over-protective parenting style increased the risk of pathological internet use [28].

Parenting style was selected as the social factor for this study is based on a) the extensive use of this predictive or risk/protective factor in the literature on adolescent addiction [29,30,31] and b) the availability of a Chinese, culturally adapted scale for evaluating parenting styles with demonstrated reliability. The scale adopted in this study evaluates three aspects of parenting style: emotional warmth (reflecting parents’ demonstrated love and care), rejection (typified by frequent punishment or strict guidance), and over-protection (otherwise defined as “taking charge”). Furthermore, since scholars have advocated for the use of family-based strategies for the prevention of online addiction [32], with recent studies particularly emphasizing parenting within the context of adolescent personality traits [31,33], the role of adolescent personality must also be considered. Thus, one potential contribution of this study is the evaluation of the effects of parenting style in conjunction with aspects of adolescents’ personality traits (discussed in the following section), a topic that has requires further empirical evaluation.

#### 1.2.2. Personality Factors: Time Management Tendency

Consideration of “time management” as a personality factor has roots in earlier research, such as that of Kelly and Johnson [34] who found a strong relationship between “time use efficiency” and the personality factor of conscientiousness. Studies on academic performance have similarly evaluated time management, specifically, as a personality measure which is strongly associated with conscientiousness, which is predictive of academic and job performance [35]. In terms of IGD, research [4,28] has found that psychological or personality traits, such as aggression and narcissism, are positively correlated with IGD, while self-control is negatively correlated with IGD. Further studies found that lower levels of conscientiousness or openness were associated with higher risk of IGD [36,37]. Thus, the role of time management in the physical and psychological health is an increasingly important topic. In fact, data from a recent randomized clinical trial of 3826 adolescents found increased screen time significantly predicts symptoms of depression [38], suggesting that the ability to manage time wisely can be beneficial to adolescents by reducing screen time and the risk of depression. Other outcomes, such as achievement and motivation, have been found to be inextricably linked to time management, since time management includes four phases of self-regulation: forethought, monitoring, control, and reflection [39]. Further research on the role of parents in time management and students’ outcomes is recommended by the author, due to the important role of parenting in developing self-regulation [39].

Autonomy and self-control are key protective personality factors in preventing or mitigating adolescents’ addiction to online games. In fact, the construct of time management has been found to be a key aspect of self-regulated learning, with researchers recommending further research into the impacts of time management on other elements of students’ performance and personal well-being [40]. As such, this research endeavors to evaluate the role of time management tendency in mediating the effects of parenting style of the increasingly serious phenomenon of IGD. Recent studies have utilized the term “time management tendency” to describe the personality trait that encapsulates elements of autonomy and self-control, attracting the attention of scholars in the field of health psychology [41,42,43]. Based on the importance of students’ sense of self-efficacy and behavioral control [19,20,21], the factor of time management may be an ideal proxy variable for predicting adolescents’ IGD. As such, according to Huang and Zhang [41], time management tendency is a stable personality trait. Time management tendency can, therefore, demonstrate an individual’s self-efficacy in accomplishing the difficult task of arranging their schedule, their self-control in avoiding engaging in time-wasting activities (which includes excessive or pathological play of online games), and their autonomy in making decisions regarding their daily activities. In fact, from the perspective of several scholars [44,45,46], the inability of individuals to properly manage their time is a risk factor for IGD. As such, we speculate that the individual-level trait of time management tendency will be predictive of a lower likelihood of IGD, as measured by pathological videogame use.

The potential of time management tendency as a mediator between social-level variables and IGD has precedent in the literature, with research demonstrating how self-efficacy, a related construct, mediates the impact of emotional intelligence (including self-management and social skills) on online gaming addiction [47]. Likewise, time management has been demonstrated to mediate the role of family-level variables, such as family stability, on attention [48]. Perceived parental autonomy support—defined as parents who encourage independent thinking, give rationales for their decisions, and acknowledge their child’s feelings—is significantly associated with developing adolescents’ self-efficacy, thereby positively impacting time management [49].

Given this increased emphasis on the interplay between personality-level variables and social or family-level variables in the prediction of Internet Gaming Disorder, our study attempts to contribute to the literature by developing a model to evaluate the potential mediating effect of time management tendency (as a personal-level, psychological variable) on the relationship between parenting style (deemed one of the most important predictors of pathological internet use) and adolescents’ IGD.

### 1.3. Research Purpose

Both the social/familial-level variable of parenting styles and the individual/personality-level variable of time management tendency have been associated with IGD. However, based on a review of the literature, there are no studies integrating the two factors of parenting styles and adolescents’ time management tendency in developing a model for predicting adolescents’ IGD. In addition, although time management tendency (reflecting an individual’s self-efficacy, self-control, and autonomy) is logically related to IGD, there is a lack of empirical evidence to directly evaluate the relationship between the two variables. Therefore, the purpose of this study was to evaluate the nature of the relationships among parenting styles, time management tendency, and adolescents’ IGD. Specifically, we proposed a mediation model. Namely, the social/familial-level variable of parenting style was predicted to have an indirect effect on IGD through the mediation of the individual-level variable of time management tendency. 

There are two reasons for hypothesizing time management tendency as a mediator. The first is that, since parenting style is related to the development of a child’s personality, parenting style should serve as an explanatory variable for personality characteristics (including time management tendency). The role of parenting style in time management tendency has been evaluated within the context of locus of control, with findings demonstrating that parental criticism, for example, is significantly correlated with procrastination (representing the lack of time management), while conscientiousness, a personality trait strongly associated with time use efficiency [34], is inversely associated with procrastination [50,51]. The second reason is that, considering that several studies suggest that addiction is strongly associated with a lack of time management [44,45,46], an individual’s time management should be more directly related to addictive behaviors, as compared to the relatively indirect influence of parenting style. Through mediation analysis, our findings can clarify the mechanisms through which parenting styles influence adolescents’ time management tendency, thereby indirectly impacting IGD. The results of this study have the potential to contribute to the development of both family education and clinical psychological counseling treatment programs, directions that have been emphasized by internet addiction studies [32] and studies evaluating parents’ role in adolescent time management [50,51].

## 2. Materials and Methods 

### 2.1. Participants

A total of 390 grade 10 and 11 students from two senior high schools in Linyi City, China were selected as participants through cluster sampling. With the assistance of the school’s head teacher, paper-based questionnaires were directly administered at the school by the research team. After excluding questionnaires with incomplete responses, the effective number of responses for statistical analysis was 357 (including 157 from boys and 200 from girls), an effective response rate of 91.5%. This study was approved by the research ethics committee of a local university in Shandong Province (IRB No. 2019QCH0307).

### 2.2. Measures

#### 2.2.1. Internet Gaming Disorder

The Pathological Video Game Use Questionnaire (PVGUQ) [52] was adopted to assess the severity of participants’ IGD. This scale was adapted from the English version [53] and has demonstrated good reliability and validity in past research with Chinese adolescents. The PVGUQ survey includes 11 items, which measure the frequency of IGD symptoms in the previous six months. Sample items include: “Do you actually spend more time playing online games than you planned to?” and “Do you feel the need to increase the amount of time you spend playing online games in order to be satisfied?”. PVGUQ items are measured on a three-point Likert-type scale: 1 (never), 2 (sometimes), and 3 (often). As such, higher PVGUQ scores indicate more serious levels of IGD. The Cronbach’s α calculated for the PVGUQ administered in this study was 0.75, indicating acceptable reliability.

#### 2.2.2. Parenting Style

A Chinese version of the Simplified Parenting Style Scale (CSPSS) [54] was adopted to measure participants’ perceptions of their parent’s parenting styles. The CSPSS is divided into three dimensions: emotional warmth (reflecting parents’ demonstrated love and care), rejection (typified by frequent punishment or strict guidance), and over-protection (otherwise defined as “taking charge”). This scale was developed specifically for Chinese students, based on Chinese parenting styles, and demonstrates strong test-retest reliability [55]. Items on the CSPPS are the same for evaluating the parenting styles of both fathers and mothers. Sample items include: “My father/mother feels proud when I succeed” (emotional warmth), “My father/mother often treats me in embarrassing ways” (rejection), and “My father/mother demands me to report what I did when I get home” (over-protection). The CSPSS was scored on a four-point Likert-type scale: 1 (Never), 2 (Occasionally), 3 (Often), and 4 (Always). Higher scores for each sub-scale indicate stronger perceptions of the adolescent regarding that parenting style. In this study, Cronbach’s α for the sub-scales were as follows emotional warmth (fathers = 0.81; mothers = 0.76); rejection (fathers = 0.80; mothers = 0.75); and over-protection (fathers = 0.81; mothers = 0.72), indicating satisfactory reliability.

#### 2.2.3. Time Management Tendency

The Time Management Tendency Scale (TMTS) developed by Huang and Zhang [41] was used to evaluate participants’ tendency towards effectively managing their time. The TMTS scale consists of 44 items measured on a five-point Likert-type scale with responses ranging from 1 (totally inconsistent) to 5 (completely consistent). Sample items from the scale include Item 1 (“I tend to arrange daily activities using a calendar”), Item 4 (“I set daily learning goals for myself”), and Item 25 (“I have a plan for what to do every week”). Higher scores on the TMTS are interpreted as a greater tendency to manage time appropriately. The factor of time management tendency is considered a personality characteristic (individual-level variable) in this study, interpreted as an adolescent’s tendency to use their time appropriately. In this study, Cronbach’s α for the scale was 0.91, which demonstrated excellent reliability.

### 2.3. Data Analysis 

Descriptive statistics were first provided for all variables and sub-scales, where applicable, (i.e., IGD, parenting styles, and time management tendency). We then computed Pearson product-moment correlation coefficients to examine the associations among the variables. In addition, Chi-squared analysis was used to compare IGD and non-IGD adolescents, categorized according to the criteria established by Yu et al. [52] and Gentile [53], in order to evaluate any differences between addicts and non-addicts in terms of the key demographic variables, including gender, grade, and whether or not the student lived in the school dormitory.

**Figure 1:**
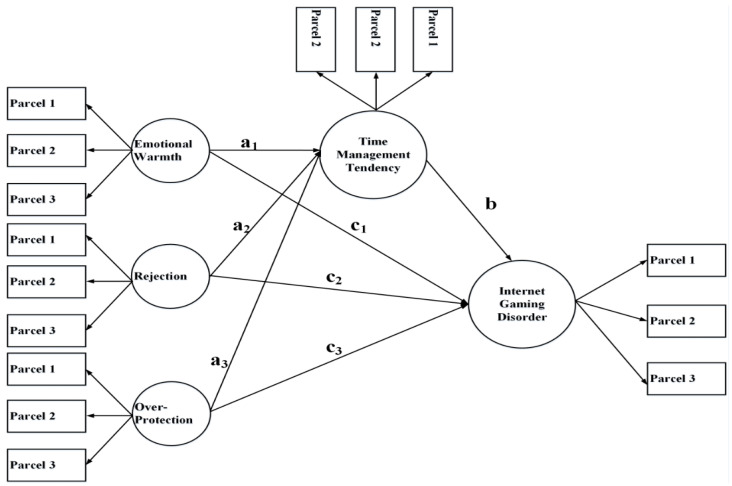
Conceptual model for mediation analysis.

Next, we conducted mediation analysis to examine whether time management tendency served as a mediator between parenting style and adolescents’ IGD. Considering the presence of significant correlations among the three parenting styles for both fathers and mothers, it was determined that the indirect effects from each of the parenting styles might cause inflated values due to shared variance with the other two styles. Thus, it was deemed inappropriate to separately analyze each effect. As such, the three parenting styles were simultaneously evaluated by mediation models for fathers and mothers separately (See Figure 1). In this way, we were able to more accurately estimate the indirect effects from parenting styles. Structural equation modeling (SEM) with LISREL 8.80 was used to test the model, and demographic variables (i.e., gender, grade, and whether or not the respondent was an only child) were included as control variables. For the measurement of the models, we adopted item parceling, with each latent variable including three parcels (due to the fact that there were more than 40 items evaluating time management tendency and 11 items measuring IGD). A combination of model fit indices was used to judge the model’s validity: comparative fit index (CFI), non-normed fit index (NNFI), incremental fit index (IFI), root mean square error of approximation (RMSEA), and standardized root mean square residual (SRMR).

After confirming the validity of the model, we followed the procedures established by Zhao et al. [56] to examine the mediation effect. First, the indirect path “a_1_ × b” was examined to check whether or not this coefficient was statistically significant (as such it was necessary to evaluate “a_2_ × b” and “a_3_ × b”). If the indirect path was not statistically significant, it was determine that there was no mediation effect. On the other hand, if the indirect path was statistically significant, then the coefficient “c” was evaluated. If “c” was not significant, the mediation type “indirect-only” was supported. If “c” was significant, then the product of “a × b × c” was evaluated as either positive or negative. If “a × b × c” was positive, this indicated “complementary mediation”; while if “a × b × c” was negative, “competitive mediation” was indicated. 

## 3. Results

### 3.1. Descriptive Statistics

As shown in Table 1, the average IGD score was 1.41 (SD = 0.41), which was lower than the median value for the scale (i.e., 2). In terms of father and mother parenting styles, the mean emotional warmth score for mothers was 2.72 (SD = 0.58) and 2.63 for fathers (SD = 0.57). These values are between “Occasionally” (2) and “Often” (3) on the sub-scale. The mean rejection score for mothers was 3.42 (SD = 0.56) and 3.50 (SD = 0.51) for fathers. These values were between “Often” (3) and “Always” (4) on the sub-scale, suggesting that, on average, adolescents evaluated their parent’s “rejection” style of parenting quite high relative to the other sub-scales. The mean over-protection score for mothers was 2.84 (SD = 0.49) and 2.99 (SD = 0.47) for fathers. These values were approaching “Often” (3) on the sub-scale, also suggesting that more negative parenting styles were generally reported by the adolescent participants. Average time management tendency was 3.28 (SD = 0.56), which was higher than the median value for the scale (i.e., 3).

### 3.2. Pearson Correlations 

In interpreting the results of the Pearson correlations, both the father’ and mothers’ emotional warmth were negatively correlated with IGD (fathers’: *r* = −0.030, *p* = 0.031; mothers’: *r* = −0.018, *p* = 0.040), suggesting that emotional warmth was associated with lower levels of online game addiction. On the other hand, a significant and positive correlation was found for both parents’ rejection and over-protection with IGD (fathers’ rejection: *r* = 0.173, *p* < 0.010; mothers’ rejection: *r* = 0.116, *p* = 0.010; fathers’ over-protection: *r* = 0.208, *p* < 0.010; mothers’ over-protection: *r* = 0.154, *p* = 0.010), suggesting that these negative parenting styles were associated with greater levels of online game addiction. Considering correlations among the three parenting styles, emotional warmth was negatively correlated with both rejection and over-protection for both fathers (rejection: *r* = −0.337, *p* < 0.010; over-protection: *r* = −0.034, *p* < 0.010) and mothers (rejection: *r* = −0.187, *p* < 0.010; over-protection: *r* = −0.137, *p* < 0.010). There was also a significant positive correlation between both parents’ emotional warmth and time management tendency (fathers: *r* = 0.243, *p* < 0.010; mothers: *r* = 0.223, *p* < 0.010), suggesting that greater emotional warmth is associated with better time management skills. On the other hand, both parents’ over-protection was negatively correlated with time management tendency (fathers’: *r* = −0.084, *p* = 0.030; mothers’: *r* = −0.119, *p* = 0.010), suggesting that these negative parenting styles are associated with lower levels of time management. Finally, time management tendency was significantly correlated negatively with IGD (*r* = −0.248, *p* < 0.010), suggesting that better time management was associated with lower levels of online game addiction.

### 3.3. Chi-Squared Analysis

As for the difference between online game addicts and non-addicts in terms of key demographic variables (see Table 2), the results demonstrated that boys were more likely to be classified as online game addicts, as compared to girls. However, the results of *χ^2^* indicated that, apart from gender, there were no significant differences between online addicts and non-addicts in terms of grade (*χ^2^* = 0.09, *p* = 0.76) and whether or not they lived in the school dormitory (*χ^2^* = 0.42, *p* = 0.52).

### 3.4. Mediation Analysis

This research focused on exploring the nature of the mediation effect of time management tendency on the relationship between parenting styles and IGD. We first examined the quality of the mediation model for fathers and mothers separately. Table 3 provides the results of model fit for the two models. From this table, we can observe that most of the indices meet the criterion that CFI and IFI are higher than 0.90; and that RMSEA and SRMR are lower than 0.09 and 0.07. However, NNFI is slightly below 0.90. Based on these results, we were able to further conduct path analysis.

**Figure 2:**
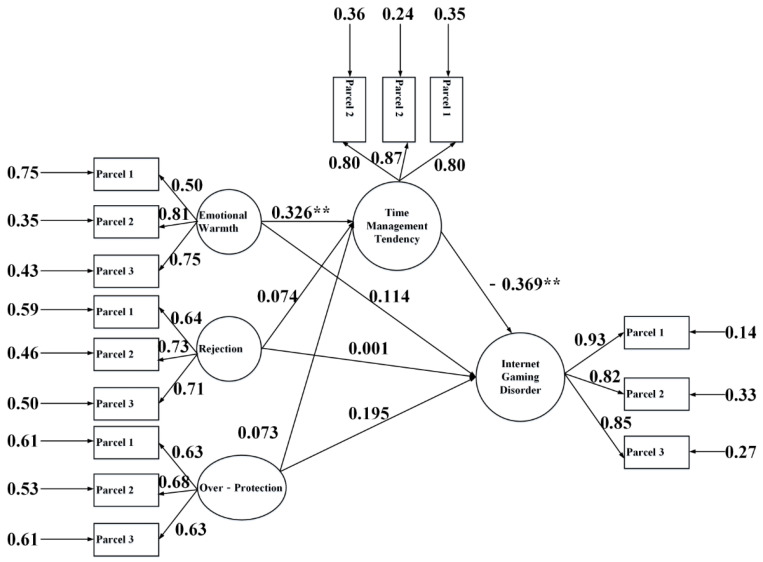
Fathers’ model for mediation analysis. Note: ** p < 0.01.

**Figure 3:**
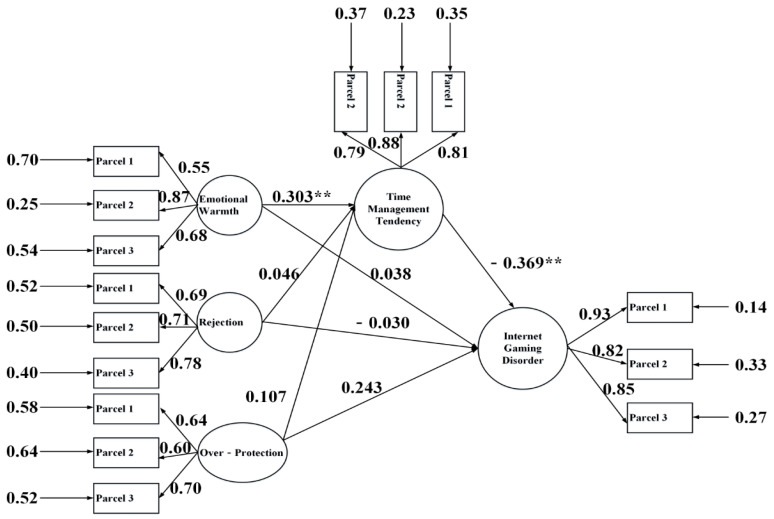
Mothers’ model for mediation analysis. Note: ** p < 0.01.

Following the procedures of Zhao et al. [56], we conducted a rigorous mediation analysis (see Figure 2 and Figure 3). The first step was to review the coefficients of the indirect path “a_1_ × b”, “a_2_ × b”, and “a_3_ × b” for both the fathers’ and mothers’ models. Results showed that the indirect effect of “a_1_ × b” (i.e., the indirect effect from emotional warmth) was significant for both parent’s models (fathers’ coefficient = −0.120, *t* = −2.657, *p* < 0.01; mothers’ coefficient = −0.112, *t* = −3.175, *p* < 0.01), while the coefficients of “a_2_ × b” and “a_3_ × b” were not significant in either model. The second step was to examine “c_1_” for the fathers’ and mothers’ models. The results indicated that “c_1_” was not significant. Therefore, according to the mediation type classification scheme proposed by Zhao et al. [42], there were only “indirect-only” types of mediation for both parents’ emotional warmth, parenting styles that indirectly alleviated IGD through the mediation of time management tendency. Finally, the explained variances were 56.0% for IGD and 19.2% for time management tendency in the fathers’ model and 56.7% for IGD and 20.7% for time management tendency in the mothers’ model.

## 4. Discussion

According to the results of this study, 44 out of a total of 357 adolescents were classified as being addicted to online games, according to the criteria of Yu et al. [52] and Gentile [53], suggesting an addiction rate of 12.3%, which is similar to the recent findings of Yang et al. [10] who reported that 13% of Chinese adolescents were addicted to online games. This number indicates that IGD is an issue that must be addressed, particularly given the well-documented negative effects of IGD on physical, psychological, and social well-being [2], as well as academic performance [1]. Descriptive analysis of parenting styles revealed that participants reported high levels for the two dimensions of fathers’ and mothers’ rejection, as well as relatively high levels of over-protective parenting styles. Furthermore, these types of parenting styles were negatively correlated with the “emotional warmth” style of parenting. Given the results of SEM analysis, wherein emotional warmth for both mothers and fathers alleviate IGD through the mediation of time management tendency, it is suggested that emotional warmth may serve as a protective factor, while over-protection (and, potentially, rejection) could be interpreted as risk factors for IGD. As such, due to the fact that only positive impacts can be found in the mediation model for emotional warmth, this is evidently the preferable parenting style in reducing internet addiction. While higher levels of more negative (over-protection or rejection) types of parenting are correlated with IGD, these are perceived as “reactive” in nature. Thus, in the mediation model (through the variable of time management tendency), these parenting styles have little impact. 

As “reactive” factors, the high reported levels of over-protection and rejection could indicate that parents recognize the seriousness of online addiction and have adopted a strict approach to prevent their children from developing IGD. Based on Chinese culture, in particular, we believe that there are two possible reasons for parents adopting strict parenting styles: the first possible reason is that high school students are facing upcoming high-stakes college entrance examinations. Therefore, as compared to junior high school or elementary school, parents hold higher expectations regarding their children’s academic performance. It is reasonable for parents to have strict parenting styles. Another possible reason is that high school students facing academic pressure may lack sufficient resilience [57], requiring parents to adopt a more strict parenting style to support them. These reasons are both consistent with the findings of scholars related to the use of “guan” [58], a concept closely associated with over-protective parenting styles and aspects of rejection as reflected in the CSPSS [55]. 

While our findings are consistent with previous studies on adolescents in that parenting styles are closely related to online addiction among adolescents [59,60,61,62], the current study adds more nuance to the manner in which parenting can impact online game addiction. In terms of parenting styles, the emotional warmth of fathers and mothers were the only aspects of parenting style associated with adolescents’ IGD, with both being associated with lower levels of adolescents’ IGD. This supports the role of “warmth” as a parenting style in serving as an effective protective factor [28,29,30,31], while further indicating that both mothers’ emotional warmth and fathers’ emotional warmth are important factors. While parental rejection and over-protection are associated with higher risk of online addiction, they were not significant in the SEM model. When each parenting style for both mothers and fathers were evaluated independently (see Appendix A), the potential partial mediation of mothers’ over-protection by time management tendency indicated a small, but potentially harmful, influence on IGD. Due to the lack of significant findings for the role of the parenting styles of rejection or over-protection, they should be cautiously considered as potential risk factors, indicating the potential of underlying issues, such as the lack of perceived self-control [19] and the resulting need for greater parental mediation [63] and online security measures [24]. 

Furthermore, the role of time management tendency as a mediator provides a clearer picture as to how different types of parenting styles can indirectly impact risk and protective factors for adolescent IGD. The finding of an indirect mediating effect of time management tendency in terms of the association of the parenting style of warmth with lower levels of IGD suggests that the social/familial-level variable of parenting style, by encouraging adolescents to engage in time management, requiring greater self-efficacy and self-control [19,20,21], can indirectly impact students’ ability to avoid the temptations of online games. Thus, as hypothesized, the role of parenting style must consider the influence of personality and individual-level variables [31,33]. These findings support Xu’s results that parents’ emotional warmth positively affects children’s time management tendency [64]. 

The results of this study also found a significant negative correlation between time management tendency and adolescents’ IGD. While these findings are consistent with previous studies [65,66], a new direction for research may be provided by focusing on the relationship between personality and IGD. Specifically, although several studies on addiction have highlighted the association between addiction and lack of time management [44,45,46], no prior studies have empirically evaluated time management as a personality trait mediating the role of other variables on the risk of IGD. While previous research has largely focused on the personality traits openness, narcissism, aggression, and extraversion [4,36,37,67], the value of self-efficacy, self-control, and autonomy [19,20,21] encapsulated by time management tendency is a contribution to the literature. Further research on the role of time management as a personality variable can further elaborate upon its role as a mediator for other social/familial-level constructs, such as peer relationships, school performance, family harmony, and emotional support [19]. 

In this study, we demonstrated the manner in which time management tendency serves as a mediator of the relationship between parenting styles and IGD. The results indicated that time management tendency indirectly mediated the influence of emotional warmth of both parents on adolescents’ IGD. The unique nature of our analytical approach in simultaneously evaluating the relationships among parenting (using parenting style as a variable), time management tendency, and online game addition contributes to a growing foundation of evidence regarding the interrelationship among family-level and personality-level variables in terms of addiction, such as IGD. More specifically, although the potential of time management tendency as a stable personality trait has attracted the attention of scholars in the field of addiction [68], few studies have considered parenting style factors in combination with time management tendency as a personality trait. The mediation model proposed in this study can more fully explain adolescents’ IGD, by integrating the factors of family support and individual personality, illustrating the application of a cognitive behavioral model, such as the approach proposed by Davis [69].

## 5. Conclusions

Our results demonstrated that approximately 13% of high school students are at risk of IGD, which indicates that the problem of online game addiction still requires further research and the development of new strategies based on a clear understanding of the mechanisms underlying both social/familial and individual/personality factors. Our mediation model including parenting style as an independent variable, time management tendency as a mediating variable, and IGD as the dependent variable has shown the benefits of parents interacting with adolescents in a warm and accepting way, in order to foster self-control, self-efficacy, and autonomy in order to engage in effective time management. 

Certain limitations of the study should be addressed. First, the use of a cross-sectional study, while informative, cannot capture the nature of the influence of parenting styles directly and indirectly on adolescents’ IGD through the development time management skills. Thus, a longitudinal model would be informative and a logical extension of this research. Another limitation is that most of our participants lived in the school dormitory (i.e., 71%), which may limit the external validity of this study. In terms of generalizability, the fact that most respondents live on campus may limit the representativeness of the sample. More specifically, since the adolescents in this study mostly live on campus, they may not interact with their parents frequently, which may limit the warmth they perceive in terms of parenting style. 

The contribution of this study to research is the extension of the paradigm of time management tendency as a personality trait encompassing self-efficacy, self-control, and autonomy, all of which can serve as protective factors for IGD. The proposed mediation model also elucidates the role of time management in mediating the influence of parents through the parenting styles evaluated in this study. Further research using other social or familial variables in order to evaluate the mediating role of time management tendency is recommended in order to expand upon this important factor in preventing the risk of online addictions. In terms of practical implications, the emphasis on parenting styles, and the evident benefit of warm parenting styles as compared to rejection and over-protection parenting styles (which may reflect some common Chinese cultural elements, such as “guan”) can assist in counselling parents of adolescents at risk of internet or online game addiction.

## Figures and Tables

**Table 1:** Descriptive statistics and Pearson correlations.

Variables	M ± SD	1	2	3	4	5	6	7
IGD	1.41 ± 0.41	1.00						
Fathers’ parenting style								
2. Emotional warmth	2.63 ± 0.57	−0.030 *	1.00					
3. Rejection	3.50 ± 0.51	0.173 *	−0.337 *	1.00				
4. Over-protection	2.99 ± 0.47	0.208 *	−0.034 *	0.460 *	1.00			
Mothers’ parenting style								
5. Emotional warmth	2.72 ± 0.58	−0.018 *	0.726 *	−0.154 *	−0.015 *	1		
6. Rejection	3.42 ± 0.56	0.116 *	−0.151 *	0.590 *	0.379 *	−0.187 *	1	
7. Over-protection	2.84 ± 0.49	0.154 *	−0.054 *	0.355 *	0.725 *	−0.137 *	0.504 *	1
8. Time management tendency	3.28 ± 0.56	−0.248 *	0.243 *	−0.065 *	−0.084 *	0.223 *	−0.013 *	−0.119 *

Note: * *p* < 0.05.

**Table 2:** Differences between addicts and non-addicts in terms of demographic variables.

Demographic Variable	Addicts (*n* = 44)	Non-Addicts (*n* = 313)	*χ^2^* (*p*-Value)
Gender			
Boys (*n* = 157)	32 (20.39%)	125 (79.61%)	16.84 (*p* < 0.01) **
Girls (*n* = 200)	12 (6.00%)	188 (94.00%)	
Grade			
10th (*n* = 286)	36 (12.58%)	250 (87.41%)	0.09 (*p* = 0.76)
11th (*n* = 71)	8 (11.27%)	63 (88.73%)	
Lives in school dormitory			
Yes (*n* = 253)	33 (13.04%)	220 (86.96%)	0.42 (*p* = 0.52)
No (*n* = 104)	11 (10.58%)	93 (89.42%)	

Note: the criterion for categorizing participants and addicts or non-addicts followed the protocols outlined in the studies of Yu et al. [52] and Gentile [53]; ** *p* < 0.01.

**Table 3:** Model fit for fathers’ and mothers’ parenting styles.

Fit Index	Fathers	Mothers
χ^2^ (df)	364.138 (110)	356.764 (110)
CFI	0.913	0.920
NNFI	0.896	0.899
IFI	0.914	0.921
RMSEA (90% CI)	0.081 (0.071–0.089)	0.079 (0.070–0.088)
SRMR	0.064	0.067

## Appendix A

When testing the mediating effect of time management tendency, we first evaluated separate models. As such, since parenting styles for both fathers and mothers included the sub-scales of emotional warmth, rejection and over-protection, a total of six models were tested (i.e., Fathers’ emotional warmth, rejection, and over-protection → time management tendency → IGD; and Mothers’ emotional warmth, rejection, and over-protection→ time management tendency→ IGD). Although evaluating each parenting style individually does not take into account the influence of the correlation among parenting styles on the mediator and dependent variables, and may result in inflated estimates, this type of analysis still has some interesting contributions. During the testing of these six models, we carefully checked the coefficients of the pathways to determine if they met the criteria to be classified as having a mediation effect with SPSS macro “PROCESS”, designed by Hayes [70].

The results revealed three significant mediation effects (see the Table A1 below): with time management tendency fully mediating the relationship between both parents’ emotional warmth and IGD; (indirect effect of fathers’ emotional warmth = −0.255, *p* < 0.001; indirect effect of mothers’ emotional warmth = −0.041, *p* = 0.010). Additionally, time management tendency partially mediated the relationship between mothers’ over-protection and IGD (Coefficient = 0.027, *p* = 0.037). While the partial mediation of mothers’ over-protection was not significant in the SEM model, this relationship might be considered as a possible risk factor impacting adolescents’ IGD.

**Table A1:** Mediating Effect of Time Management Tendency.

The Path	Indirect Effect Coefficient	Standard Error	95% Confidence Interval			Mediation
			Lower Bounds	Upper Bounds	*p*	
1. Father’s rejection → time management tendency → online game addiction	−0.014	0.013	−0.042	0.010	0.217	none
2. Father’s emotional warmth → time management tendency → online game addiction	−0.255	0.068	−0.421	−0.143	<0.001 *	full
3. Father’s over-protection → time management tendency → IGD	0.020	0.013	−0.003	0.052	0.133	none
4. Mother’s rejection → time management tendency → IGD	−0.003	0.011	−0.025	0.018	0.798	none
5. Mother’s emotional warmth → time management tendency → IGD	−0.041	0.012	−0.069	−0.021	0.010 *	full
6. Mother’s over-protection → time management tendency → IGD	0.027	0.012	0.004	0.053	0.037 *	partial

Note: * *p* < 0.05.

## References

[1] Block J.J. (2008). Issues for DSM-V: Internet addiction. Am. J. Psychiatry.

[2] Nakayama H., Mihara S., Higuchi S. (2017). Treatment and risk factors of Internet use disorders. Psychiatry Clin. Neurosci..

[3] Kim K., Kim K. (2015). Internet game addiction, parental attachment, and parenting of adolescents in South Korea. J. Child Adolesc. Subst. Abuse..

[4] Kim E.J., Namkoong K., Ku T., Kim S.J. (2008). The relationship between online game addiction and aggression, self-control and narcissistic personality traits. J. Eur. Psychiatry.

[5] Sublette V.A., Mullan B. (2012). Consequences of play: A systematic review of the effects of online gaming. Int. J. Ment. Health Addict..

[6] Eichenbaum A., Kattner F., Bradford D., Gentile D.A., Choo H., Chen V.H.H., Khoo A., Green C.S. (2015). The role of game genres and the development of internet gaming disorder in school-aged children. J. Addict. Behav. Ther. Rehabil..

[7] Rehbein F., Kliem S., Baier D., Mößle T., Petry N.M. (2015). Prevalence of internet gaming disorder in German adolescents: Diagnostic contribution of the nine DSM-5 criteria in a state-wide representative sample. Addiction.

[8] China Internet Network Information Center Statistical Report on China’s Internet Development. http://www.cac.gov.cn/2019-02/28/c_1124175677.htm.

[9] Long J., Liu T., Liu Y., Hao W., Maurage P., Billieux J. (2018). Prevalence and correlates of problematic online gaming: A systematic review of the evidence published in Chinese. Curr. Addict. Rep..

[10] Yang X., Jiang X., Mo P.H., Cai Y., Ma L., Lau J.F. (2020). Prevalence and interpersonal correlates of internet gaming disorders among Chinese adolescents. Int. J. Environ. Res. Public Health.

[11] Lin C.Y., Imani V., Broström A., Årestedt K., Pakpour A.H., Griffiths M.D. (2019). Evaluating the psychometric properties of the 7-item Persian Game Addiction Scale for Iranian adolescents. Front. Psychol..

[12] Karaca S., Karakoc A., Can Gurkan O., Onan N., Barlas G.U. (2020). Investigation of the online game addiction level, sociodemographic characteristics and social anxiety as risk factors for online game addiction in middle school students. Community Ment. Health J..

[13] Stevens M.W., Dorstyn D., Delfabbro P.H., King D.L. (2020). Global prevalence of gaming disorder: A systematic review and meta-analysis. Aust. N. Z. J. Psychiatry.

[14] Zheng C.X., Ofir T., Yu F.Y. (2012). Online game addiction among adolescents: Motivation and prevention factors. Eur. J. Inform. Syst..

[15] Li H., Wang S. (2013). The role of cognitive distortion in online game addiction among Chinese adolescents. J. Child. Youth Serv. Rev..

[16] Choi C., Hums M.A., Bum C.H. (2018). Impact of the family environment on juvenile mental health: ESports online game addiction and delinquency. Int. J. Environ. Res. Public Health.

[17] Xinhua News Agency, Game Supervision Starting from Classification. https://xhpfmapi.zhongguowangshi.com/vh512/share/9266815?channel=weixin&from=timeline.

[18] People’s Daily Game Addiction, Can This Disease Be Cured?. http://paper.people.com.cn/rmrbhwb/html/2020-09/07/node_865.htm.

[19] Mihara S., Higuchi S. (2017). Cross-sectional and longitudinal epidemiological studies of Internet gaming disorder: A systematic review of the literature. Psychiatry Clin. Neurosci..

[20] Berte D.Z., Mahamid F.A., Affouneh S. (2019). Internet addiction and perceived self-efficacy among university students. Int. J. Ment. Health Addict..

[21] Chung S.J., Jang J.H., Lee J.Y., Choi A., Kim B.M., Park M.K., Jung M.H., Choi J.S. (2020). Self-efficacy and clinical characteristics in casual gamers compared to excessive gaming users and non-gamers in young adults. J. Clin. Med..

[22] Chou H.L., Chou C., Chen C.H. (2016). The moderating effects of parenting styles on the relation between the internet attitudes and internet behaviors of high-school students in Taiwan. Comput. Educ..

[23] Lin C.H., Lin S.L., Wu C.P. (2009). The effects of parental monitoring and leisure boredom on adolescents’ internet addiction. Adolescence.

[24] Floros G.D., Siomos K.E., Fisoun V., Dafouli E., Geroukalis D. (2013). Adolescent online cyberbullying in Greece: The impact of parental online security practices, bonding, and online impulsiveness. J. School Health.

[25] Huang X.Q., Zhang H.M., Li M.C., Wang J.N., Zhang Y., Tao R. (2010). Mental health, personality, and parental rearing styles of adolescents with internet addiction disorder. Cyberpsychol. Behav. Soc. Netw..

[26] Siomos K., Floros G., Fisoun V., Evaggelia D., Farkonas N., Sergentani E., Lamprou M., Geroukalis D. (2012). Evolution of internet addiction in Greek adolescent students over a two-year period: The impact of parental bonding. Eur. Child Adolesc. Psychiatry.

[27] Li S., Lei H., Tian L. (2018). A meta-analysis of the relationship between parenting style and internet addiction among mainland Chinese teenagers. Soc. Behav. Personal..

[28] Ni X., Qian Y., Wang Y. (2017). Factors affecting pathological internet use among Chinese university students. Soc. Behav. Personal..

[29] Karaer Y., Akdemir D. (2019). Parenting styles, perceived social support and emotion regulation in adolescents with internet addiction. Compr. Psychiatry.

[30] Floros G., Siomos K. (2013). The relationship between optimal parenting, Internet addiction and motives for social networking in adolescence. Psychiatry Res..

[31] Zhang R.P., Bai B.Y., Jiang S., Yang S., Zhou Q. (2019). Parenting styles and internet addiction in Chinese adolescents: Conscientiousness as a mediator and teacher support as a moderator. Comput. Hum. Behav..

[32] Wu C.S.T., Wong H.T., Yu K.F., Fok K.W., Yeung S.M., Lan C.H. (2016). Parenting approaches, family functionality, and internet addiction among Hong Kong adolescents. BMC Pediatr..

[33] Sun Y., Wilkinson J.S. (2020). Parenting style, personality traits, and interpersonal relationships: A model of prediction of internet addiction. Int. J. Commun. U. S..

[34] Kelly W.E., Johnson J.L. (2005). Time use efficiency and the five-factor model of personality. Education.

[35] Butter R., Born M.P. (2012). Enhancing criterion-related validity through bottom-up contextualization of personality inventories: The construction of an ecological conscientiousness scale for Ph. D. candidates. Hum. Perform..

[36] Wang C.W., Ho R.T.H., Chan C.L.W., Tse S. (2015). Exploring personality characteristics of Chinese adolescents with internet-related addictive behaviors: Trait differences for gaming addiction and social networking addiction. Addict. Behav..

[37] Wittek C.T., Finserås T.R., Pallesen S., Mentzoni R.A., Hanss D., Griffiths M.D., Molde H. (2016). Prevalence and predictors of video game addiction: A study based on a national representative sample of gamers. Int. J. Ment. Health Addict..

[38] Boers E., Afzali M.H., Newton N., Conrod P. (2019). Association of screen time and depression in adolescence. JAMA Pediatr..

[39] Xu J., Du J., Wang C., Liu F., Huang B., Zhang M., Xie J. (2020). Intrinsic motivation, favorability, time management, and achievement: A cross-lagged panel analysis. Learn. Motiv..

[40] Wolters C.A., Won S., Hussain M. (2017). Examining the relations of time management and procrastination within a model of self-regulated learning. Metacognition Learn..

[41] Huang X.T., Zhang Z.J. (2001). The compiling of adolescence time management disposition inventory. Acta Psychol. Sin..

[42] Liu W., Zhang S.S., An L. (2011). The measurement of college students’ time management tendency and its relationship to culture orientation. Psychol. Sci..

[43] Yan Y., Sun L., Feng K. (2018). The mediating effect of college students’ time management tendency on the relation between fatigue and test anxiety. China J. Health Psychol..

[44] Anand V. (2007). A study of time management: The correlation between video game usage and academic performance markers. CyberPsychol. Behav..

[45] Chiu S.I., Lee J.Z., Huang D.H. (2004). Video game addiction in children and teenagers in Taiwan. J. CyberPsychol. Behav..

[46] Wood R.T. (2008). Problems with the concept of video game “addiction”: Some case study examples. Int. J. Ment. Health Addict..

[47] Che D., Hu J., Zhen S., Yu C., Li B., Chang X., Zhang W. (2017). Dimensions of emotional intelligence and online gaming addiction in adolescence: The indirect effects of two facets of perceived stress. Front. Psychol..

[48] Malatras J.W., Israel A.C., Sokolowski K.L., Ryan J. (2016). First things first: Family activities and routines, time management and attention. J. Appl. Dev. Psychol..

[49] Won S., Shirley L.Y. (2018). Relations of perceived parental autonomy support and control with adolescents’ academic time management and procrastination. Learn. Individ. Differ..

[50] Boysan M., Kiral E. (2017). Associations between procrastination, personality, perfectionism, self-esteem and locus of control. Br. J. Guid. Couns..

[51] Shih S.S. (2017). Factors related to Taiwanese adolescents’ academic procrastination, time management, and perfectionism. J. Educ. Res..

[52] Yu C., Li X., Zhang W. (2015). Predicting adolescent problematic online game use from teacher autonomy support, basic psychological needs satisfaction, and school engagement: A 2-year longitudinal study. Cyberpsychol. Behav. Soc. Netw..

[53] Gentile D. (2009). Pathological video-game use among youth ages 8 to 18: A national study. Psychol. Sci..

[54] Jiang G., Lu Z.R., Jiang B.J., Xu Y. (2010). Preliminary revision of the Chinese version of the parenting style questionnaire. Xīn Lǐ Fā Zhǎn Yǔ Jiāo Yù.

[55] Duan Y. (2016). Parenting Styles, Effortful Control, and Academic Outcomes among Chinese Adolescents: The Mediating Effect of Activation Control. Master’s Thesis.

[56] Zhao X., Lynch J.G., Chen Q. (2010). Reconsidering baron and kenny: Myths and truths about mediation analysis. J. Consum. Res..

[57] Chen X., Cheung H.Y., Fan X., Wu J. (2018). Factors related to resilience of academically gifted students in the Chinese cultural and educational environment. Psychol. Sch..

[58] Lan X., Scrimin S., Moscardino U. (2019). Perceived parental guan and school adjustment among Chinese early adolescents: The moderating role of interdependent self-construal. J. Adolesc..

[59] Abedini Y., Zamani B.E., Kheradmand A., Rajabizadeh G. (2012). Impacts of mothers’ occupation status and parenting styles on levels of self-control, addiction to computer games, and educational progress of adolescents. Addict. Health.

[60] Květon P., Jelínek M. (2016). Parenting styles and their relation to videogame addiction. Int. J. Psychol. Behav. Sci..

[61] Özgur H. (2019). Online game addiction among Turkish adolescents: The effect of internet parenting style. Malays. Online J. Educ. Technol..

[62] Zandi Payam A., Mirzaeidoostan Z. (2019). Online game addiction relationship with cognitive distortion, parenting style, and narcissistic personality traits in students. Iran. J. Psychiatry Clin. Psychol..

[63] Chang F.C., Chiu C.H., Miao N.F., Chen P.H., Lee C.M., Chiang J.T., Pan Y.C. (2015). The relationship between parental mediation and internet addiction among adolescents, and the association with cyberbullying and depression. Compr. Psychiatry.

[64] Xu L. (2017). Research on the correlation between college students’ time management tendency and their parenting style. Hēi Lóng Jiāng Gāo Jiāo Yán Jiù.

[65] Peng H.L., Jiang X.Y. (2011). Relationship between internet addiction and time management tendency of college students. Zhōng Guó Gōng Gòng Wèi Shēng.

[66] Jiao Y. (2019). Effects of time management on college students’ mobile phone addiction. Adv. Psychol..

[67] Andreassen C.S., Griffiths M.D., Gjertsen S.R., Krossbakken E., Kvam S., Pallesen S. (2013). The relationships between behavioral addictions and the five-factor model of personality. J. Behav. Addict..

[68] He C., Xia M., Jiang G.R., Wei H. (2012). Self-esteem and internet game addiction-the mediating role of self-control. Zhōng Guó Lín Chuáng Xīn Lǐ Xué.

[69] Davis R.A. (2001). A cognitive-behavioral model of pathological internet use. Comput. Hum. Behav..

[70] Hayes A.F. (2013). Methodology in the Social Sciences. Introduction to Mediation, Moderation, and Conditional Process Analysis: A Regression-Based Approach.

